# Intramedullary Headless Screw Feasibility for Anatomical Reduction in II–V Metacarpal Fractures: A CT-Based Morphometric Study

**DOI:** 10.3390/jcm15093468

**Published:** 2026-05-01

**Authors:** Pelin İsmailoğlu, Cengiz Kazdal, Emrehan Uysal, Alp Bayramoğlu

**Affiliations:** 1 Department of Anatomy, Faculty of Medicine, Recep Tayyip Erdogan University, 53020 Rize, Turkey; pelin.ismailoglu@erdogan.edu.tr; 2 Department of Orthopaedics and Traumatology, Faculty of Medicine, Recep Tayyip Erdogan University, 53020 Rize, Turkey; cengiz.kazdal@erdogan.edu.tr; 3 Faculty of Medicine, Recep Tayyip Erdogan University, 53020 Rize, Turkey; emrehan_uysal24@erdogan.edu.tr; 4 Department of Anatomy, Faculty of Medicine, Acibadem Mehmet Ali Aydinlar University, 34752 Istanbul, Turkey

**Keywords:** metacarpal intramedullary canal, metacarpal headless screw, morphometric analysis, virtual simulation

## Abstract

**Background and Objectives**: Intramedullary headless screw (IMHS) fixation is a minimally invasive and biomechanically stable option for metacarpal fractures. However, the suitability of commonly used screw diameters may be limited by the morphometric features of the intramedullary canal. This study evaluated the isthmus morphology of the second to fifth metacarpals using computed tomography (CT)-based morphometric analysis and virtual screw simulation. **Materials and Methods**: A retrospective morphometric study was conducted using 75 hand CT scans, representing 300 metacarpals (second to fifth). Three-dimensional reconstructions were created with Mimics software (Materialise, Leuven, Belgium), and the isthmus level was identified by serial axial CT analysis. Canal diameters were measured at this level, and bone-specific virtual screw models were generated in Rhinoceros 3D and imported into Mimics for virtual implantation and canal conformity assessment. Feasibility rates were calculated for screw diameters between 2.75 mm and 4.00 mm. The effects of age and gender were also analyzed. **Results**: The fourth metacarpal had the smallest mean isthmus diameter (2.64 ± 0.89 mm), while the fifth had the largest (3.21 ± 0.84 mm). Feasibility decreased as screw diameter increased across all metacarpals. The fourth metacarpal showed the lowest compatibility, with feasibility rates of 10.7% for 3.5 mm screws and 4.0% for 4.0 mm screws. In contrast, the fifth metacarpal had the highest feasibility at smaller diameters, with 74.7% compatibility for 2.75 mm screws and 62.7% for 3.0 mm screws. Positive correlations were found between age and isthmus diameters of the second and third metacarpals, indicating age-related canal widening. **Conclusions**: The anatomical feasibility of IMHS fixation in the second to fifth metacarpals is influenced by isthmus morphology. The fourth metacarpal appears to be the most restrictive, particularly for screws ≥ 3.5 mm. These findings support individualized CT-based preoperative templating rather than standardized implant selection to improve screw canal compatibility and reduce cortical compromise risk.

## 1. Introduction

Metacarpal fractures account for a significant proportion of all hand and forearm injuries, representing between 18% and 41% of cases [1,2]. Metacarpals are generally considered to play a critical role in hand function by serving as attachment sites for intrinsic hand muscles and forming the structural arch that connects the digits to the carpus [3,4]. These injuries predominantly affect young, active individuals, particularly males, often resulting from sports-related trauma, falls, or direct impact [5,6]. Even relatively minor deformities, such as angulation, malrotation, or a shortening of just 2 mm, have the potential to impair hand biomechanics, leading to reduced grip strength, tendon imbalance, and limited range of motion [3]. Therefore, restoration of anatomical alignment is often viewed as a key goal in management to preserve optimal hand function, as a detailed understanding of normal dimensions is essential for guiding surgical reconstruction in cases of malunion or traumatic bone loss [1,4].

The limitations of traditional surgical options for unstable fractures, such as Kirschner wire fixation and dorsal plating, are well documented [7,8]. Although K-wires are minimally invasive, they may fail to provide rigid fixation, often requiring prolonged immobilization and carrying a risk of pin tract infection or hardware migration [9]. Conversely, dorsal plates provide rigid stability but require extensive soft tissue dissection, which is frequently associated with extensor tendon adhesions, hardware prominence, and joint stiffness [10]. Intramedullary headless screw (IMHS) fixation has emerged over the past decade as an effective surgical option for the management of displaced metacarpal fractures [7]. Initially developed as a practical solution to overcome the limitations of conventional methods, this technique provides both stability and preservation of soft tissue function [3,7]. Since its introduction as a retrograde technique for subcapital fractures, this method has gained increasing popularity due to its potential for relative technical simplicity, its ability to provide stable fixation of unstable patterns, and the reduction in operative time compared with conventional methods [7]. Furthermore, the minimally invasive nature of intramedullary screw fixation facilitates early mobilization and rapid functional recovery, with reported complication rates comparable to those of alternative fixation strategies [6,11].

Further biomechanical evidence supports this approach, suggesting that fixation with an intramedullary headless screw provides sufficient stability to withstand the repeated forces associated with an early range of motion [5,12]. Intramedullary headless screw fixation is a minimally invasive technique that provides adequate rotational stability through isthmic canal fit while minimizing soft tissue disruption [7,13]. However, the success of this technique may be significantly influenced by the anatomical compatibility between the implant and the medullary canal [14]. It is generally suggested that successful intramedullary fixation may depend more on accurate anatomical templating than on implant design alone [7]. Intramedullary screw fixation relies on achieving rotational stability through an optimal fit within the narrowest segment of the medullary canal, the isthmus. Therefore, the isthmus may be a critical limiting factor for safe screw placement [14,15]. Although three-dimensional (3D) computed tomography (CT) data suggests using an articular starting point for these extra-articular fractures, there are still concerns about causing iatrogenic damage to the articular surface. Studies show that retrograde insertion usually results in the loss of between 4% and 12% of the cartilaginous head surface [15,16].

Metacarpal morphology is complex and varies significantly between individuals, specific digits, and biological factors such as age or gender [17]. While previous studies have evaluated metacarpal morphology and canal dimensions, further detailed 3D assessment of the isthmus with direct clinical applicability to screw feasibility could provide additional clarity [4,18]. Conventional radiographs may occasionally be insufficient for accurate morphometric evaluation of metacarpals due to overlapping bony structures, particularly in the lateral view [1,4,9]. Computed tomography (CT) enables a more precise three-dimensional (3D) assessment of metacarpal anatomy, thereby facilitating improved surgical planning and potentially more accurate implant selection [4,17,18]. Furthermore, establishing reliable morphometric reference values may contribute to improved anatomical restoration in cases of traumatic bone loss or surgical reconstruction [4]. The intrinsic curvature and angular morphology of metacarpal bones may also complicate the selection of an optimal surgical approach and the safe execution of intramedullary fixation techniques [17]. Accurate identification of the intramedullary canal axis and the determination of an appropriate entry point are therefore considered critical to minimize articular surface damage and attempt to achieve stable fixation [15,17]. Previous CT-based studies have attempted to define optimal entry locations, showing that antegrade insertion might be performed with minimal violation of the articular surface when guided by 3D assessment, while retrograde access might be achieved by systemizing the joint surface geometry [15,18].

In the present study, the narrowest segments of the second through fifth metacarpals were first evaluated in the sagittal, coronal, and axial planes to accurately identify and mark the specific isthmus level for each individual metacarpal. Subsequently, the widest intramedullary canal diameter was measured at this identified isthmus location to define clinically relevant parameters that may facilitate safer and more anatomically accurate fracture fixation [17]. An optimal isthmic fit is widely considered essential for successful intramedullary headless screw fixation, making accurate evaluation of canal morphology and achieving an “interference fit” key factor in proper implant selection [14,15]. This study was designed as a morphometric analysis with a clinical focus, aiming to provide practical guidance on selecting intramedullary screws by assessing the clinical applicability of anatomical findings. Therefore, in order to assess the theoretical feasibility of accommodating various commonly used screw diameters, ranging from 2.75 mm to 4.00 mm, we aimed to evaluate the intramedullary canal morphometry. This study aims to provide quantitative morphometric observations that may serve as a supplemental reference for clinicians by characterizing intramedullary isthmus diameters and exploring the potential influence of age and gender. These findings could inform preoperative templating and hardware selection, potentially enabling more precise anatomical considerations in the management of contemporary metacarpal fractures.

## 2. Materials and Methods

This study was designed as a retrospective, clinically oriented morphometric study based on three-dimensional computed tomography. The study protocol was approved by the Clinical Research Ethics Committee of Acıbadem University and Acıbadem Healthcare Institutions (ATADEK; approval number: 2026-04/158) and was conducted in accordance with the principles of the Declaration of Helsinki. Prior to evaluation, the institutional radiology service fully anonymized all imaging data and associated demographic records, ensuring that no identifiable patient information was accessible to the researchers during the analysis period.

**Figure 1:**
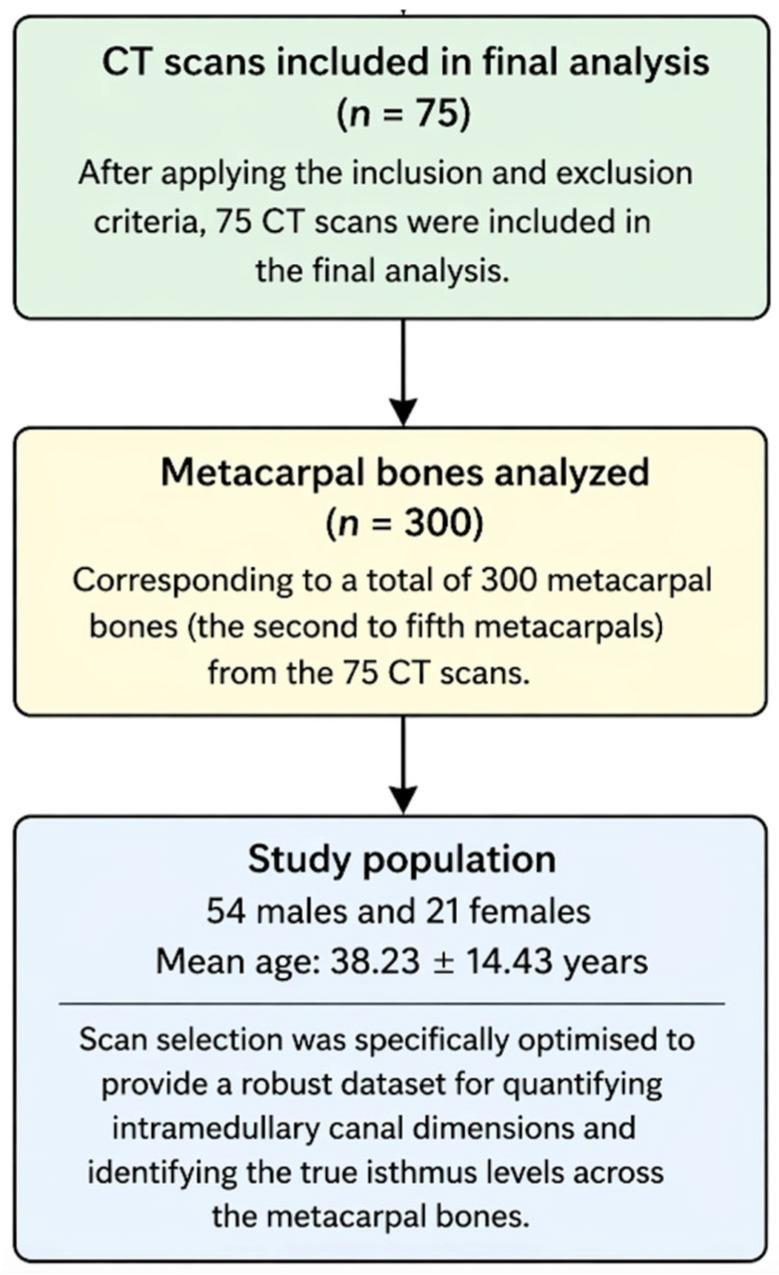
Flow diagram of CT scan selection and inclusion process. A total of 350 hand and wrist CT scans were initially assessed for eligibility. After applying predefined exclusion criteria—including trauma, prior surgery or hardware, congenital deformities, degenerative or inflammatory bone pathology, osseous defects, and inadequate image quality—scans were excluded. The remaining 75 CT scans were included in the final analysis, corresponding to 300 metacarpal bones (second to fifth). The final study cohort consisted of 54 males and 21 females, with a mean age of 38.23 ± 14.43 years.

A total of 350 hand CT scans, obtained from the institutional radiology archive, were consecutively screened for eligibility. Scans were included if the second to fifth metacarpals were fully visualized and anatomically intact, and of sufficient quality for multiplanar and three-dimensional reconstruction. Exclusion criteria included acute or previous hand trauma; metacarpal or phalangeal fractures; prior hardware or surgery involving the hand; congenital skeletal deformities; inflammatory or degenerative bone pathology; osseous defects; and imaging artifacts that could interfere with an accurate morphometric assessment. After applying these criteria, 75 CT scans were included in the final analysis, corresponding to a total of 300 metacarpal bones (the second to fifth metacarpals). The study population consisted of 54 males and 21 females, with a mean age of 38.23 ± 14.43 years. Scan selection was specifically optimized to provide a robust dataset for quantifying intramedullary canal dimensions and identifying the true isthmus levels across the metacarpal bones. As this study was based on retrospective CT imaging data, detailed clinical variables such as indications for imaging, comorbidities, or treatment-related parameters were not available. The study is summarized in a flow diagram (Figure 1).

All CT imaging was performed using a Somatom Force multidetector CT system (Siemens Healthineers, Erlangen, Germany). The acquisition protocol was standardized with a tube voltage of 100 kVp, a tube current of 256 mAs, and an image matrix resolution of 512 × 512 pixels. Scans were reconstructed using a standard bone algorithm with a slice thickness of 0.5 to 1.0 mm, resulting in an approximate voxel size of 0.7 × 0.7 × 0.5 mm^3^. A field of view (FOV) between 160 and 180 mm was used to include the entire hand, allowing complete visualization of the second to fifth metacarpals. The CT datasets were subsequently converted into three-dimensional (3D) models using Mimics^®^ software (Materialise, Leuven, Belgium). These 3D reconstructions were used for detailed morphometric and morphological evaluations, as well as virtual screw feasibility analysis. The applied imaging parameters provided high resolution multiplanar and three-dimensional reconstructions necessary for millimetric precision throughout the assessment process.

Two stages of quality control procedures were performed. Firstly, during image selection, only high-quality CT scans that preserved cortical and intramedullary anatomical detail were included in the study. Secondly, during the reconstruction and measurement phase, scans that presented segmentation difficulties, incomplete metacarpal visualization, or technical issues that would affect morphometric assessment were excluded prior to the final analysis. Consequently, there were no measurement failures in the final dataset. All morphometric measurements and virtual screw simulations were performed by two independent, blinded observers. Observer 1, who had eight years of clinical experience in hand pathology, completed the initial set of measurements and repeated the entire analysis approximately two months later to assess intraobserver reliability. Observer 2 (with 10 years’ experience in musculoskeletal morphology) independently repeated all measurements in a blinded manner to evaluate interobserver reliability. As this study was based on retrospective CT imaging data, no surgical procedures were performed as part of the study design, and variables related to operating surgeons or intra-operator differences were not applicable.

### 2.1. Measurement Technique and Virtual Screw Simulation

**Figure 2:**
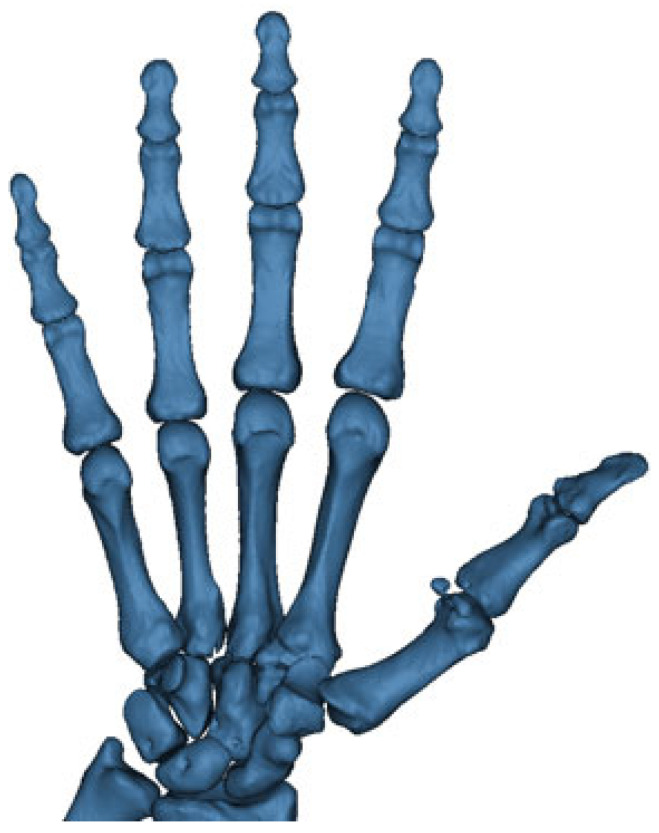
Three-dimensional reconstruction of hand computed tomography data generated in Mimics^®^ software (Materialise, Leuven, Belgium). The second to fifth metacarpals were identified.

Following three-dimensional reconstruction of the hand models in Mimics^®^ software (Materialise, Leuven, Belgium), a standardized morphometric and virtual simulation workflow was applied for each of the second through fifth metacarpals. Initially, the complete osseous anatomy of the hand was reconstructed in three dimensions, and the second through fifth metacarpals were individually identified and isolated for subsequent analysis (Figure 2).

**Figure 3:**
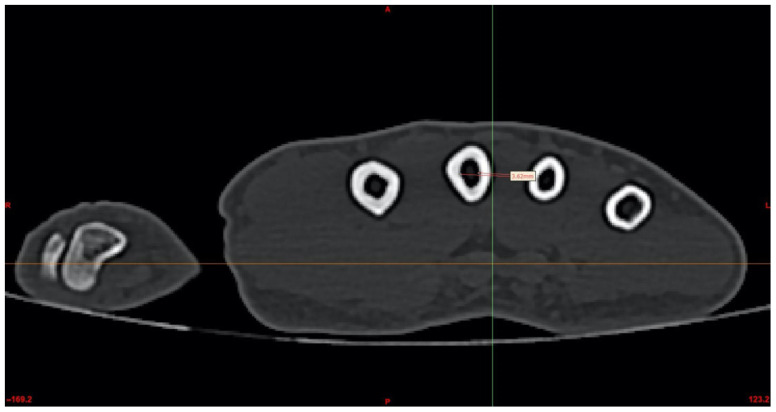
Measurement of the level of the isthmus and the intramedullary canal. The isthmus level of the third metacarpal bone was identified and measured, along with the axial localization of the isthmus level and the intramedullary canal. In the example shown, the diameter of the third metacarpal isthmus measured 3.62 mm.

The intramedullary canal of each metacarpal was then evaluated using serial axial CT sections to determine the narrowest portion of the canal, corresponding to the true isthmus level. Representative axial localization of the isthmus is demonstrated in Figure 3.

**Figure 4:**
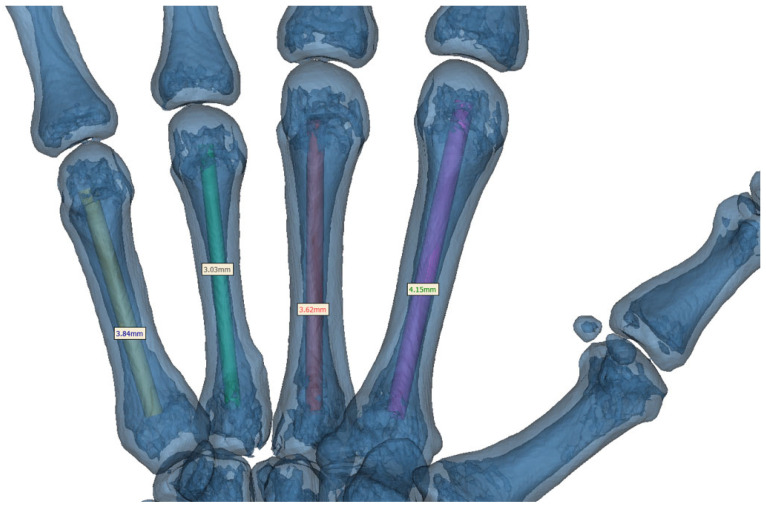
Transparent three-dimensional reconstruction demonstrating STL-based virtual screw simulation. STL screw models generated in Rhinoceros 3D software were imported into Mimics^®^ software and virtually inserted into each metacarpal according to the metacarpal-specific dimensions summarized in Table 1. For each metacarpal, the screw was positioned to achieve an anatomically compatible isthmus level fit within the intramedullary canal and to demonstrate canal conformity on transparent three-dimensional bone reconstruction.

For each of the second through fifth metacarpals, the true isthmus level was first identified and the intramedullary canal diameter was measured at this level. Based on these metacarpal-specific measurements, a corresponding screw with an anatomically compatible diameter was subsequently designed in Rhinoceros 3D software (version 8.0 Robert McNeel & Associates, Seattle, WA, USA) and virtually inserted into the metacarpal canal for three-dimensional feasibility analysis. For example, in the representative axial section of the third metacarpal shown in Figure 3, the intramedullary diameter at the isthmus level was measured as 3.62 mm; accordingly, a 3.0 mm diameter virtual screw was created in Rhinoceros 3D software and subsequently inserted for simulation (Figure 4).

The measured isthmus diameters and the corresponding metacarpal-specific virtual screw dimensions are summarized in Table 1. Colored cylindrical screw models were subsequently designed in Rhinoceros 3D software according to the predefined dimensions summarized in Table 1. The screw diameter allocation for each metacarpal is illustrated in Figure 4. Following the importation of the STL file into Mimics^®^ software, the placement of the intramedullary screw was visualized using a transparent, three-dimensional bone reconstruction. This clearly demonstrated the screw’s trajectory and relationship with the canal within each metacarpal (Figure 4).

Based on the measured canal diameters (Table 1), metacarpal isthmus-specific virtual screw sizes were selected to reflect feasible intramedullary fixation while maintaining an appropriate cortical clearance margin. To simulate realistic surgical implant selection, screw diameters were intentionally chosen to be slightly smaller than the measured isthmus diameters.

**Figure 5:**
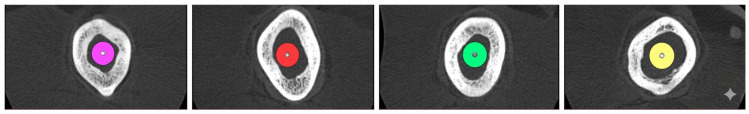
Axial cross-sectional measurement of intramedullary isthmus diameters. Representative axial cross-sectional images demonstrating intramedullary canal diameter measurements at the respective isthmus level of the second through fifth metacarpals (MC). Purple = MC2, red = MC3, green = MC4, yellow = MC5.

Subsequently, an axial cross-sectional reassessment was performed at the isthmus level of each metacarpal following virtual placement to determine whether the simulated screw fit of the intramedullary canal appropriately and maintained canal conformity. Final post-implantation axial verification is shown in Figure 5.

**Figure 6:**
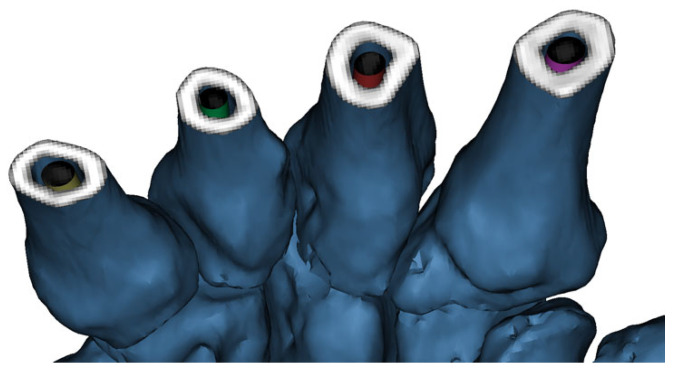
Proximal entry site planning and screw size allocation. Three-dimensional proximal view of the second through fifth metacarpals demonstrating the planned entry zones and metacarpal-specific screw size allocation prior to virtual intramedullary insertion.

To further illustrate the planned proximal entry zones and metacarpal-specific screw size allocation, a three-dimensional proximal view of the second through fifth metacarpals was generated prior to virtual insertion (Figure 6). This view was used to visually confirm the intended entry points and the anatomical compatibility of the selected screw diameters before intramedullary placement.

**Figure 7:**
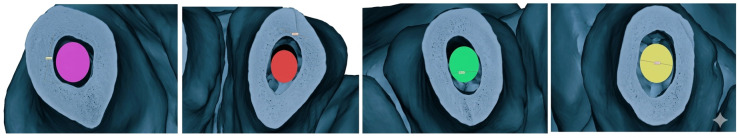
Three-dimensional verification of screw fit at the isthmus level after virtual insertion. Representative three-dimensional sectional images demonstrating screw fit and canal conformity at the respective isthmus level of the second through fifth metacarpals (MCs) after virtual simulation. Purple = MC2, red = MC3, green = MC4, yellow = MC5.

Following virtual placement, three-dimensional sectional reassessment was performed at the respective isthmus level of each metacarpal to determine whether the simulated screw appropriately fit within the intramedullary canal and maintained canal conformity (Figure 7).

Definition of feasibility: The study workflow consisted of two complementary components. First, feasibility analysis was performed across the entire dataset using a morphometric threshold approach based on the relationship between the measured intramedullary isthmus diameter and predefined screw diameters. Specifically, anatomical feasibility was defined as a condition in which the isthmus diameter was greater than the screw diameter (isthmus diameter > screw diameter). This quantitative criterion was applied consistently to all metacarpals to determine the proportion capable of accommodating each screw size.

Second, three-dimensional virtual screw simulations were conducted on representative metacarpals to illustrate canal conformity, screw trajectory, and anatomical fit. These simulations served as a qualitative validation and visualization tool rather than the computational basis for feasibility calculations. In this context, virtual implantation was used to confirm the absence of apparent cortical breach and to support the anatomical plausibility of the morphometric findings. From a biomechanical perspective, it is acknowledged that optimal fixation requires preservation of a minimal cortical wall thickness to reduce the risk of iatrogenic fracture. However, due to interindividual variability in bone quality and the absence of universally accepted cutoff values for minimum cortical thickness in metacarpals, a fixed numerical clearance margin was not imposed.

### 2.2. Statistical Analysis

All statistical analyses were performed using IBM SPSS Statistics software version 26.0 (IBM Corp., Armonk, NY, USA), and a *p* value of < 0.05 was considered statistically significant for all analyses. Prior to the main analyses, an a priori sample size calculation was performed based on previously published metacarpal morphometric data. Specifically, the effect size used for the G*Power analysis was derived from the morphometric measurements reported by Örs et al., in which the proximal width of the second metacarpal demonstrated a statistically significant difference between male and female participants (16.69 ± 1.72 mm vs. 15.27 ± 1.33 mm, respectively; *p* < 0.001) [17]. Based on these values, Cohen’s d effect size was calculated as 0.92. Using this effect size, an a priori power analysis was conducted with G*Power software (version 3.1.9.7), assuming an independent samples *t* test, a one-tailed hypothesis, a significance level of α = 0.05, a statistical power of 95% (1 − β = 0.95), and equal group allocation (N2/N1 = 1). According to this analysis, a minimum of 27 cases per group, corresponding to a total sample size of 54 participants, was considered sufficient to ensure adequate statistical power.

Descriptive statistics were presented as mean ± standard deviation for continuous variables. The distributional characteristics of the data were evaluated using the Shapiro–Wilk test, supported by visual inspection of histograms and assessment of skewness and kurtosis values. Variables demonstrating approximate normality were analyzed using parametric methods. Comparisons of intramedullary isthmus diameters between male and female participants were performed using independent samples *t* tests, and effect sizes were reported as Cohen’s d. For age-based comparisons, participants were stratified into three predefined age categories (<30 years, 31–50 years, and ≥51 years), and differences among groups were analyzed using one-way analysis of variance (ANOVA). Homogeneity of variances was assessed using Levene’s test prior to ANOVA. When statistically significant differences were identified, Tukey’s honestly significant difference (HSD) test was used for post hoc pairwise comparisons to account for multiple testing and control the family-wise error rate. The magnitude of group differences was expressed using eta squared (η^2^). Associations between age and intramedullary canal dimensions, as well as interrelationships among metacarpal isthmus measurements, were evaluated using Pearson correlation analysis. Correlation coefficients were reported together with Holm adjusted *p* values where appropriate. Measurement reproducibility was assessed using the intraclass correlation coefficient (ICC) based on a two-way mixed effects model with absolute agreement. Both single-measure and average-measure ICC values were calculated to evaluate intraobserver and interobserver reliability.

## 3. Results

A total of 75 hand CT scans, corresponding to 300 metacarpal bones (from the second to the fifth metacarpal), were included in the final analysis. The study population consisted of 54 men and 21 women, with an average age of 38.23 ± 14.43 years. Overall, measurable variability in intramedullary isthmus diameters was observed across the metacarpals, with the fourth metacarpal exhibiting relatively smaller dimensions and the third and fifth metacarpals exhibiting relatively larger dimensions. Further feasibility analysis demonstrated a progressive decline in anatomical compatibility with increasing screw diameter, emphasizing the importance of an individualized preoperative morphometric assessment. Detailed statistical findings are presented below.

To determine the most appropriate statistical approach for the morphometric comparisons, the distributional properties of age and intramedullary isthmus diameter measurements were first evaluated. Normality analyses were performed using the Shapiro–Wilk test, together with visual inspection of distributional shape and assessment of skewness and kurtosis values. The normality assessment demonstrated that all variables were approximately normally distributed except for the fifth metacarpal isthmus diameter, which showed a statistically significant deviation from normality (W = 0.96, *p* = 0.019). However, the skewness and kurtosis values remained within acceptable limits for approximate normality, supporting the use of parametric comparisons where appropriate [19,20]. Descriptive statistics are presented in Table 2. Among the evaluated metacarpals, the fourth metacarpal demonstrated the smallest mean isthmus diameter, whereas the fifth metacarpal showed the largest mean diameter, indicating substantial anatomical variability across the metacarpal canal system (Table 2).

Independent-samples *t* tests were conducted to compare intramedullary isthmus diameters between female and male participants across the second to fifth metacarpals. No statistically significant gender-based differences were observed for any metacarpal measurement. Effect size estimates were uniformly small (Cohen’s d = −0.01 to −0.27), indicating minimal differences between groups. Detailed numerical results are presented in Table 3.

### Feasibility Analysis of Intramedullary Screw Diameters

Three-dimensional virtual screw simulation demonstrated that the patient-specific STL screw models remained centrally confined within the intramedullary canal of the second to fifth metacarpals when aligned along the anatomical canal axis. The fourth metacarpal showed the most limited intramedullary tolerance, whereas the fifth metacarpal allowed the widest simulated screw trajectory. These observations visually supported the morphometric feasibility findings obtained from axial isthmus measurements.

Feasibility analyses were performed to determine the proportion of metacarpals capable of accommodating commonly used intramedullary headless screw diameters ranging from 2.75 mm to 4.00 mm. A clear diameter-dependent reduction in anatomical compatibility was observed across all metacarpals. For the second metacarpal, feasibility progressively decreased from 52.0% at 2.75 mm to 17.3% at 4.00 mm. The third metacarpal demonstrated the highest overall feasibility profile, with 64.0% compatibility at 2.75 mm, decreasing to 17.3% at 4.00 mm. In contrast, the fourth metacarpal consistently showed the lowest anatomical feasibility, with only 46.7% compatibility at 2.75 mm, 10.7% at 3.50 mm, and 4.0% at 4.00 mm. The fifth metacarpal showed the most favorable feasibility at lower diameters, with 74.7% compatibility at 2.75 mm and 62.7% at 3.00 mm, although this also declined substantially at larger screw sizes. Overall, increasing screw diameter was associated with a marked reduction in anatomical compatibility, particularly in the fourth metacarpal, supporting the importance of individualized preoperative morphometric templating (Table 4).

Pearson correlation analysis was performed to evaluate the relationship between age and intramedullary isthmus diameters of the second to fifth metacarpals, as well as the interrelationships among metacarpal canal dimensions. A significant positive correlation was observed between age and the second metacarpal isthmus diameter (r = 0.30, *p* = 0.029), indicating that older age was associated with a larger second metacarpal canal diameter. Similarly, age was positively correlated with the third metacarpal isthmus diameter (r = 0.32, *p* = 0.022). No statistically significant correlations were found between age and the fourth (r = 0.23, *p* = 0.094) or fifth metacarpal isthmus diameters (r = 0.21, *p* = 0.094). Strong positive correlations were observed among all metacarpal isthmus measurements themselves. The strongest association was identified between the second and third metacarpals (r = 0.86, *p* < 0.001), followed by the third and fourth metacarpals (r = 0.84, *p* < 0.001). These findings suggest that individuals with a wider canal in one metacarpal tend to demonstrate proportionally wider intramedullary canals in adjacent metacarpals (Table 5).

To further explore age-related morphometric variation, one-way ANOVA was performed to compare intramedullary isthmus diameters across three predefined age categories (younger than 30 years, 31–50 years, and 51 years and older). The assumption of homogeneity of variances was satisfied for all metacarpal measurements based on Levene’s test (all *p* > 0.05), supporting the use of parametric group comparisons. No significant heterogeneity of variance was detected across groups. This indicates that variance differences across age groups were unlikely to bias the observed mean comparisons. A statistically significant difference was identified for the second metacarpal isthmus diameter across age categories (F(2,72) = 3.72, *p* = 0.029, η^2^ = 0.094), indicating a small-to-moderate age effect. Mean values and group-specific descriptive statistics are presented in Table 6. Mean values demonstrated a progressive increase across age groups, rising from 2.79 ± 1.08 mm in participants younger than 30 years to 2.70 ± 0.91 mm in those aged 31–50 years, and reaching 3.48 ± 0.93 mm in participants aged 51 years and older. This pattern suggests that age-related canal widening may become more pronounced after the age of 50. Post hoc Tukey HSD analysis revealed that the ≥51 years group had a significantly larger second metacarpal isthmus diameter compared with the 31–50 years group (mean difference = 0.78 mm, 95% CI 0.08 to 1.48, *p* = 0.024). This pairwise difference indicates that the oldest age group primarily drove the overall ANOVA significance. The comparison between the ≥51 years and <30 years groups showed a similar directional increase but did not reach statistical significance (mean difference = 0.69 mm, 95% CI −0.11 to 1.49 *p* = 0.096). Although non-significant, the consistent directionality supports the presence of a broader age-associated widening trend. For the third metacarpal, the omnibus ANOVA approached significance (F(2,72) = 2.96, *p* = 0.058, η^2^ = 0.076), with mean canal diameter increasing from 3.03 ± 1.10 mm (<30 years) and 3.05 ± 0.88 mm (31–50 years) to 3.71 ± 0.97 mm (≥51 years), suggesting a similar but less robust age-related widening pattern. This near-significant finding may reflect a parallel anatomical aging process that did not reach sufficient statistical strength in the current sample. However, Tukey post hoc comparisons did not identify statistically significant pairwise differences. This suggests that the observed trend was distributed gradually across groups rather than being driven by a single age contrast. No statistically significant age category differences were observed for the fourth metacarpal (F(2,72) = 1.39, *p* = 0.255, η^2^ = 0.037) or the fifth metacarpal (F(2,72) = 1.13, *p* = 0.329, η^2^ = 0.030). These findings suggest that age-related variation may be less pronounced in the more ulnar metacarpals. Nevertheless, both metacarpals demonstrated numerically larger mean isthmus diameters in the oldest age group, consistent with the broader age-related widening trend observed in the second and third metacarpals (Table 6). Taken together, these results indicate that age-related canal enlargement may preferentially affect the radial metacarpals.

Measurement reproducibility was assessed using the intraclass correlation coefficient (ICC) based on a two-way mixed-effects model with absolute agreement. For the intraobserver reliability analysis, repeated measurements obtained by the same observer after a 1-month interval demonstrated good agreement, with a single-measure ICC of 0.761 (95% CI: 0.669–0.834, *p* < 0.001). The corresponding average-measures ICC was 0.962 (95% CI: 0.942–0.976), indicating excellent reproducibility when repeated measurements were averaged. For the interobserver reliability analysis, independent measurements obtained by a second observer also demonstrated good agreement, with a single-measure ICC of 0.753 (95% CI: 0.660–0.828, *p* < 0.001). The average-measures ICC was 0.961 (95% CI: 0.939–0.975), again indicating excellent consistency across observers. Taken together, these findings demonstrate that the morphometric measurement protocol yielded highly reproducible intramedullary isthmus assessments across time and between observers, supporting the methodological robustness of the study (Table 7).

## 4. Discussion

Intramedullary headless screw fixation has been described as a significant advancement in the management of metacarpal fractures, potentially offering a reduction in soft tissue complications compared with traditional plating techniques [1,7,17]. However, observations from the present study suggest that the practical implementation of this technique might be influenced by the inherent anatomical characteristics of the metacarpal isthmus [4,9,14]. The principal finding of the present study is that the fourth metacarpal represents the most anatomically restrictive intramedullary canal, whereas the fifth metacarpal demonstrates the widest canal dimensions. These findings support the view that successful intramedullary fixation depends not only on the design of the implant, but also on meticulous anatomical planning and appropriate screw selection [1,7].

Feasibility analyses of this study determined the proportion of metacarpals capable of accommodating commonly used screw diameters ranging from 2.75 mm to 4.00 mm. A clear diameter-dependent reduction in anatomical compatibility was observed across all metacarpals. For the second metacarpal, feasibility progressively decreased from 52.0% at 2.75 mm to 17.3% at 4.00 mm. In contrast, the fourth metacarpal consistently showed the lowest anatomical feasibility, with only 4.0% compatibility at 4.00 mm. This marked decline in compatibility with increasing screw diameter highlights that anatomical feasibility, rather than implant availability alone, may represent the principal limiting factor in clinical application. These results align with previous research identifying the ring finger as the narrowest digit in the metacarpal cascade [4,9,18]. Furthermore, the positive correlation between age and the isthmus diameters of the second and third metacarpals indicates a propensity for age-related canal enlargement, particularly in the radial metacarpals. These findings are consistent with those of Dunleavy et al., who reported that older patients had significantly larger medullary canal diameters across the metacarpal bones [9]. In our cohort, the significant increase in the diameter of the second metacarpal isthmus after the age of 50 supports the idea that age-related cortical remodeling can paradoxically make it easier to place larger diameter screws in older patients than in younger individuals, whose canals are narrower and denser. This is likely to be a consequence of age-related cortical thinning and endosteal remodeling, whereby bone is progressively laid down at the periphery, thereby increasing the internal dimensions of the canal [9,21]. While younger patients with narrower canals are at a higher risk of iatrogenic fracture if an oversized screw is forced into the isthmus, older patients with more osteopenic bone and wider canals may actually accommodate larger-diameter screws more easily [9,14]. As emphasized by Allen et al., the degree of intramedullary canal narrowing is a critical determinant of construct stability; therefore, the age-related expansion observed in the radial metacarpals may allow clinicians to utilize more robust hardware in older populations to achieve adequate endosteal purchase and rotational stability [12,22].

Morphometric analysis of the metacarpal intramedullary canal largely aligns with previous studies. Notably, our identification of the fourth metacarpal as having the narrowest mean isthmus diameter (2.64 ± 0.89 mm) is consistent with the findings of Reddy et al., who reported a minimum intramedullary diameter of 2.65 ± 0.63 mm in the same digit [4]. Other studies have also identified the ring finger as the narrowest metacarpal. For example, Dunleavy et al. found this to be the case in both men and women, and Hoang et al. (2021) likewise reported the ring metacarpal to have the smallest medullary width in CT-based analyses [9,18]. These observations, which are consistent across different cohorts and imaging approaches, reinforce the reproducibility of our findings and the view that the fourth metacarpal is the most anatomically restrictive canal for intramedullary fixation. Similarly, the relatively wider canal dimensions observed in the fifth metacarpal in our cohort (3.21 ± 0.84 mm) are consistent with previous reports. Okoli et al. and Dunleavy et al. both described the small finger metacarpal as having the most accommodating medullary canal dimensions [1,9]. While our data showed that the third metacarpal had the second widest canal, previous work by Örs et al. indicated that this digit may have the widest cavity in certain imaging planes [17]. Differences in measurement methodology may also be partially related to this observation. While Reddy et al. identified the narrowest canal diameter at the predefined midshaft level, the present study aimed to identify the minimal isthmus diameter by systematically reviewing axial CT slices in conjunction with multiplanar reconstruction [4]. Due to the three-dimensional geometry and curvature of the metacarpal canal, a three-dimensional CT-based approach may provide a more comprehensive assessment of canal morphology and may offer more detailed anatomical information than studies based on two-dimensional measurements obtained from CT images or conventional radiographs.

The findings of the present study suggest that appropriate screw selection in the management of metacarpal fractures should not be based solely on implant characteristics or the range of commercially available screw diameters; rather, detailed evaluation of the underlying intramedullary canal morphology appears to be equally important for achieving anatomical compatibility, stable fixation, and proper anatomical reduction. Although various screw sizes are commercially available, our feasibility analysis indicates that screw diameters of between 2.75 and 3.0 mm are likely to be possible in most cases. Compatibility rates were found to range from 46.7% to 74.7% for 2.75 mm screws and from 34.7% to 62.7% for 3.0 mm screws across the metacarpal bones. However, using screw diameters of 3.5–4.0 mm may pose a particular risk, especially for the fourth metacarpal, where the feasibility rate dropped substantially to 10.7% and 4.0%, respectively. These results are consistent with those of Graf et al., who observed that, while larger-diameter screws can greatly improve construct stability, their clinical use is strictly limited by the narrowest part of the medullary canal to prevent iatrogenic cortical damage [23]. Furthermore, our data suggest that standard screw selection may not be appropriate for the second to fifth metacarpals due to substantial anatomical differences. Successful intramedullary fixation appears to depend heavily on implant–bone interactions, with the construct functioning as an internal splint that requires precise endosteal fit, as highlighted in previous reports [7]. Forcing an oversized implant into the narrow isthmus of the ring finger, which is consistently identified as the narrowest digit, may increase the risk of implant fixation or iatrogenic fracture [4,9,18]. Consequently, individualized preoperative templating using advanced three-dimensional (3D) imaging is recommended to ensure an optimal “interference fit” and to enhance the mechanical stability of the fixation [1,15]. This perspective is further supported by Thomas et al., who emphasized that meticulous anatomical planning is crucial for minimizing technical challenges and optimizing patient-specific outcomes in the management of contemporary metacarpal fractures [24]. Based on these findings, it is evident that anatomical compatibility should be considered alongside implant availability when selecting the appropriate screw diameter for metacarpal fracture fixation.

A biomechanical perspective reveals that the morphometric findings of this study could offer valuable anatomical insights to inform future biomechanical research. Current biomechanical evidence indicates that larger screw diameters and fully threaded designs can significantly enhance construct stability, failure load and torsional strength [5,14,25]. According to existing evidence, the relationship between the screw and canal diameters is a key factor in determining construct stability [5,12]. Larger screw diameters are associated with greater bending stiffness than alternative fixation methods, such as plates or wires [5,12]. Similarly, research by Chiu et al. suggested that headless compression screws can provide up to 25.4% greater resistance to maximum fracture forces compared to locked plates [10]. However, a key finding of our feasibility analysis is that implants that are preferred from a mechanical perspective are not always anatomically feasible. While larger implants may offer potential biomechanical advantages, our findings indicate that increasing screw diameter can significantly reduce anatomical compatibility. For instance, in our study group, only 4.0% of fourth metacarpals were compatible with a 4.00 mm screw. This underscores the warning by Graf et al. that forcing an oversized implant into a narrow canal risks iatrogenic cortical compromise [23]. These observations imply that anatomical limitations, especially at the endosteal isthmus, are a significant practical barrier that determines the biomechanical effectiveness of intramedullary fixation. Therefore, integrating detailed morphological information may assist with operative planning and implant selection, as also suggested in previous reports [26]. In this context, this study defines the anatomical limits of compatibility between the screw and the intramedullary canal across the metacarpal bone. It may therefore serve as a supplemental reference for future biomechanical models.

In terms of intramedullary isthmus diameters, no statistically significant gender-based differences were observed in the present sample. This observation is in line with previous reports indicating that, although external bone dimensions such as length and cortical width are generally greater in males, internal medullary canal dimensions appear to remain relatively similar between genders [4,17]. Similarly, Dunleavy et al. found no significant gender-related differences in the narrowest point of the canal, reinforcing the perspective that implant diameter selection should be guided by individual canal morphology rather than patient gender [9]. However, given the relatively small number of female participants and the small observed effect sizes, the present study may be underpowered to detect subtle gender-related differences in canal morphology, and these findings should therefore be interpreted with caution. In contrast to the uniformity across genders, age appeared to be a significant determinant of canal dimensions in specific digits. Our analysis revealed a significant positive correlation between age and the isthmus diameters of the second and third metacarpals, indicating a tendency toward age-related canal widening in these metacarpals. Specifically, the mean isthmus diameter of the second metacarpal was significantly larger in participants aged 51 years and older (3.48 mm) compared to those aged 31–50 years (2.70 mm). This finding is likely related to cortical remodeling and age-related endosteal resorption, a process in which the medullary space gradually increases as the surrounding cortex becomes thinner with advancing age [9,21]. These findings have clinical implications, suggesting that age-related canal widening may allow larger diameter screws to be used in older patients, potentially enhancing construct stability [4,5,27]. Although younger patients often have narrower, denser canals that restrict the size of screws and increase the risk of iatrogenic fracture, the expansion of the isthmus observed in our older cohort, particularly in the second and third metacarpals, can accommodate larger implants [9,27,28]. Previous reports suggest that age-related expansion of the intramedullary canal, despite a potential reduction in bone quality, may facilitate the use of larger implants in older patients, provided that cortical margins are carefully preserved [4,5,9,27,29]. Overall, the age-related widening observed in the intramedullary canal may contribute to the improved feasibility of larger screw diameters in older patients.

The observations from this study reveal that a thorough three-dimensional morphometric evaluation is crucial for achieving the precision necessary for stable fixation with headless intramedullary screws. In this context, stable fixation may be facilitated by achieving appropriate implant fit within the narrowest segment of the medullary canal, namely the isthmus, which may help support rotational stability and bending stiffness [1,7,12]. However, our results demonstrate significant anatomical variation within the metacarpal bones, with mean isthmus diameters ranging from 2.64 ± 0.89 mm for the fourth metacarpal to 3.21 ± 0.84 mm for the fifth metacarpal. These differences at such a fine scale suggest that even minor discrepancies in implant selection could affect the risk of iatrogenic cortical compromise or inadequate endosteal purchase [5]. The current literature suggests that conventional two-dimensional (2D) radiographs may be inadequate for precise planning due to overlapping bony structures, particularly in lateral views. This can lead to underestimation or overestimation of native dimensions [1,4]. As highlighted by Örs et al. (2022), metacarpals possess irregular geometric properties that differ significantly between the sagittal and coronal planes, making 3D assessment essential for characterizing the entire endosteal volume [17]. Furthermore, defining the optimal entry point and trajectory through a 3D morphometric approach is often viewed as critical for minimizing iatrogenic damage to the articular surface [4,16]. Research by Berg et al. (2013) and Bachoura et al. (2023) indicates that retrograde insertion typically compromises only about 4% to 5.6% of the cartilaginous head surface when guided by precise dorsal-third entry points [15,16]. Clinically, integrating these 3D observations might serve as a supplemental reference for individualized preoperative templating. As Allen et al. and Heilig et al. have demonstrated, the screw-to-canal diameter ratio is a fundamental determinant of construct stability and load transfer [5,12,22]. In this context, while our study is primarily morphometric rather than biomechanical, the high degree of precision afforded by 3D evaluation may help clinicians better anticipate anatomical constraints and optimize the mechanical environment for fracture healing [5,11,27,30]. Ultimately, these findings suggest that three-dimensional morphometric evaluation might enhance the predictability and safety of contemporary metacarpal fracture management [17]. Overall, these findings support the potential role of three-dimensional morphometric evaluation in achieving the anatomical precision required for stable fixation.

### Limitations

Several limitations of the present study should be acknowledged. First, the retrospective design based on an existing radiographic database may introduce a degree of selection bias and does not account for potential variability related to soft tissue conditions or population-specific anatomical differences. Second, the study focused on theoretical anatomical feasibility derived from three-dimensional CT measurements rather than direct physical implementation, and therefore may not fully reflect the technical challenges encountered during actual intramedullary screw insertion. From a clinical and biomechanical standpoint, it should be emphasized that a simple diameter-based feasibility definition does not fully capture the complexity of implant–bone interactions. Preservation of a sufficient cortical envelope is critical to avoid iatrogenic fracture, and, therefore, the results of this study should be interpreted as an anatomical screening rather than a direct surgical recommendation.

In addition, the absence of cadaveric or experimental validation remains an important limitation, as direct testing would be required to determine whether the predicted implant and canal compatibility can be achieved without cortical compromise or implant incarceration. The absence of a comparative control group represents a limitation of the present study design. Future studies incorporating comparative groups, such as pathological versus normal metacarpals or different population cohorts, may further strengthen the clinical applicability of these findings. Furthermore, as this investigation was designed as a morphometric study, clinical outcome measures such as fracture union, grip strength, range of motion, or patient-reported functional scores were not available. Accordingly, the relationship between anatomical compatibility and postoperative functional outcomes could not be directly assessed. In addition, clinical parameters such as indications for imaging, comorbidities, hospital stay, and operative variables were not available in the retrospective dataset and could not be evaluated. Future studies incorporating cadaveric validation, biomechanical testing, and prospective clinical correlation may help further clarify the relationship between screw to canal compatibility and fixation stability as well as its potential clinical implications. Additionally, as this was a retrospective imaging-based study, hand dominance information was not available and could not be evaluated. Only unilateral CT scans were included for each participant; therefore, bilateral comparisons could not be performed. Although this approach ensured statistical independence between observations, it may limit the assessment of potential functional or dominance-related asymmetries in metacarpal canal morphology. The study was also conducted at a single center, reflecting a relatively homogeneous patient population. As metacarpal morphometry may vary across different ethnic and geographic populations, the findings of this study may not be fully generalizable to other populations. Future studies including more diverse, multicenter cohorts may help to clarify population-based anatomical variability further.

## 5. Conclusions

In summary, while intramedullary headless screw (IMHS) fixation may represent an important advancement in the management of displaced metacarpal fractures by providing stable fixation through a minimally invasive approach, its clinical applicability appears to be closely influenced by the internal morphometry of the medullary canal. The findings of the present study suggest that the fourth metacarpal may represent the most anatomically restrictive segment, demonstrating the narrowest mean isthmus diameter (2.64 ± 0.89 mm) and markedly reduced feasibility for screws measuring 3.5 mm or larger. Conversely, the age-related medullary expansion observed in the second and third metacarpals suggests that older patients may accommodate larger implants more readily. Taken together, these findings support the potential value of individualized, millimetric preoperative templating rather than reliance on standardized implant selection alone. An important contribution of the present study is that it helps bridge the gap between anatomical morphology and clinically relevant implant selection. Ultimately, three-dimensional CT-based evaluation may serve as a useful adjunct in guiding implant selection according to native bone morphology and anatomical variability across the metacarpal bones.

## Figures and Tables

**Table 1:** Values are derived from a single representative case selected for illustrative three-dimensional simulation and do not reflect mean morphometric measurements.

Metacarpal	Isthmus Diameter (mm)	Virtual Screw Size Diameter (mm)
MC2 (purple in Figure 4)	4.15	3.5
MC3 (red in Figure 4)	3.62	3.0
MC4 (green in Figure 4)	3.03	2.75
MC5 (yellow in Figure 4)	3.84	3.2

**Table 2:** Baseline morphometric characteristics and normality testing of intramedullary isthmus diameters.

Variable	Mean ± SD	W	*p*	Skewness	Kurtosis
Age	38.23 ± 14.43	0.97	0.099	0.34	−0.44
2nd metacarpal isthmus	2.88 ± 1.00	0.98	0.174	0.01	−0.68
3rd metacarpal isthmus	3.17 ± 0.98	0.98	0.318	0.48	0.74
4th metacarpal isthmus	2.64 ± 0.89	0.99	0.604	0.27	0.26
5th metacarpal isthmus	3.21 ± 0.84	0.96	0.019	0.74	1.71

**Table 3:** Gender-based comparison of intramedullary isthmus diameters across the second to fifth metacarpals.

Variable	Female (*n* = 21) Mean ± SD	Male (*n* = 54) Mean ± SD	t (df)	*p*	Cohen’s d
2nd metacarpal isthmus	2.68 ± 1.01	2.95 ± 0.99	−1.04 (73)	0.303	−0.27
3rd metacarpal isthmus	3.17 ± 0.87	3.18 ± 1.03	−0.04 (73)	0.971	−0.01
4th metacarpal isthmus	2.49 ± 0.88	2.70 ± 0.90	−0.94 (73)	0.349	−0.24
5th metacarpal isthmus	3.05 ± 0.68	3.26 ± 0.90	−0.97 (73)	0.337	−0.25

**Table 4:** Feasibility rates of intramedullary headless screw diameters across the second to fifth metacarpals.

Screw Diameter	2nd MC Feasible *n* (%)	3rd MC Feasible *n* (%)	4th MC Feasible *n* (%)	5th MC Feasible *n* (%)
2.75 mm	39 (52.0)	48 (64.0)	35 (46.7)	56 (74.7)
3.00 mm	33 (44.0)	41 (54.7)	26 (34.7)	47 (62.7)
3.50 mm	23 (30.7)	27 (36.0)	8 (10.7)	22 (29.3)
4.00 mm	13 (17.3)	13 (17.3)	3 (4.0)	12 (16.0)

**Table 5:** Pearson correlation matrix for age and intramedullary isthmus diameters.

Variable	1	2	3	4	5
**1. Age**	—				
**2. 2nd MC isthmus**	0.30 *p* = 0.029	—			
**3. 3rd MC isthmus**	0.32 *p* = 0.022	0.86 *p* < 0.001	—		
**4. 4th MC isthmus**	0.23 *p* = 0.094	0.80 *p* < 0.001	0.84 *p* < 0.001	—	
**5. 5th MC isthmus**	0.21 *p* = 0.094	0.73 *p* < 0.001	0.81 *p* < 0.001	0.78 *p* < 0.001	—

Note: Statistically significant correlations are shown in bold. Pearson correlation coefficients are presented with Holm-adjusted *p* values.

**Table 6:** One-way ANOVA comparison of intramedullary isthmus diameters across age categories.

Variable	<30 Years Mean ± SD	31–50 Years Mean ± SD	≥51 Years Mean ± SD	F(2,72)	*p*	η^2^
**2nd MC isthmus**	2.79 ± 1.08	2.70 ± 0.91	**3.48 ± 0.93**	**3.72**	**0.029**	**0.094**
**3rd MC isthmus**	3.03 ± 1.10	3.05 ± 0.88	3.71 ± 0.97	2.96	0.058	0.076
**4th MC isthmus**	2.60 ± 1.04	2.54 ± 0.77	2.98 ± 0.96	1.39	0.255	0.037
**5th MC isthmus**	3.22 ± 0.77	3.10 ± 0.80	3.48 ± 1.02	1.13	0.329	0.030

Note: Statistically significant findings are shown in bold. Tukey post hoc testing showed a significant difference only between the 31–50 years and ≥51 years groups for the second metacarpal (*p* = 0.024).

**Table 7:** Intraobserver and interobserver reliability of intramedullary isthmus measurements.

Reliability Type	Single-Measure ICC (95% CI)	Average-Measure ICC (95% CI)	*p*
Intraobserver (1-month repeat)	0.761 (0.669–0.834)	0.962 (0.942–0.976)	<0.001
Interobserver (independent observer)	0.753 (0.660–0.828)	0.961 (0.939–0.975)	<0.001

## Data Availability

We confirm that the main data supporting this study’s findings are available within the article, and any additional data are available upon request from the corresponding author.

## Abbreviations

The following abbreviations are used in this manuscript:

MC	Metacarpal
3D	Three-dimensional
CT	Computed tomography
